# Insights into Cold-Season Adaptation of Mongolian Wild Asses Revealed by Gut Microbiome Metagenomics

**DOI:** 10.3390/microorganisms13102304

**Published:** 2025-10-04

**Authors:** Jianeng Wang, Haifeng Gu, Hongmei Gao, Tongzuo Zhang, Feng Jiang, Pengfei Song, Yan Liu, Qing Fan, Youjie Xu, Ruidong Zhang

**Affiliations:** 1 Key Laboratory of Adaptation and Evolution of Plateau Biota, Northwest Institute of Plateau Biology, Chinese Academy of Sciences, Xining 810001, China; wangjianeng@nwipb.cas.cn (J.W.); guhaifeng@nwipb.cas.cn (H.G.); gaohm@nwipb.cas.cn (H.G.); zhangtz@nwipb.cas.cn (T.Z.); jiangfeng@nwipb.cas.cn (F.J.); pfsong@nwipb.cas.cn (P.S.); 2 College of Life Sciences, University of Chinese Academy of Sciences, Beijing 100049, China; 3 Qinghai Provincial Key Laboratory of Animal Ecological Genomics, Xining 810001, China; 4 College of Life Science and Technology, Inner Mongolia Normal University, Hohhot 010022, China; liuyan@imnu.edu.cn; 5 Urat Middle Banner Management Station of National Nature Reserve of Haloxylon Ammodendron and Equus Hemionus, Bayannur 015300, China; fanqing202508@163.com; 6 Urad Rear Banner Management Station of National Nature Reserve of Haloxylon Ammodendron and Equus Hemionus, Bayannur 015543, China; 13904783490@163.com

**Keywords:** Mongolian wild ass, seasonal adaptation, cold-season survival, gut microbiota, metagenomics, trnL-based plant composition

## Abstract

The Mongolian wild ass (*Equus hemionus hemionus*) is a flagship species of the desert-steppe ecosystem in Asia, and understanding its strategies for coping with cold environments is vital for both revealing its survival mechanisms and informing conservation efforts. In this study, we employed metagenomic sequencing to characterize the composition and functional potential of the gut microbiota, and applied DNA metabarcoding of the chloroplast trnL (UAA) g–h fragment to analyze dietary composition, aiming to reveal seasonal variations and the interplay between dietary plant composition and gut microbial communities. In the cold season, Bacteroidota and Euryarchaeota were significantly enriched, suggesting enhanced fiber degradation and energy extraction from low-quality forage. Moreover, genera such as *Bacteroides* and *Alistipes* were also significantly enriched and associated with short-chain fatty acid (SCFA) metabolism, bile acid tolerance, and immune modulation. In the cold season, higher Simpson index values and tighter principal coordinates analysis (PCoA) clustering indicated a more diverse and stable microbiota under harsh environmental conditions, which may represent an important microecological strategy for the host to cope with extreme environments. Functional predictions based on the Kyoto Encyclopedia of Genes and Genomes (KEGG) further indicated upregulation of metabolic and signaling pathways, including ABC transporters, two-component systems, and quorum sensing, suggesting multi-level microbial responses to low temperatures and nutritional stress. trnL-based plant composition analysis indicated seasonal shifts, with Tamaricaceae detected more in the warm season and Poaceae, Chenopodiaceae, and Amaryllidaceae detected more in the cold season. Correlation analyses revealed that dominant microbial phyla were associated with the degradation of fiber, polysaccharides, and plant secondary metabolites, which may help maintain host energy and metabolic homeostasis. Despite the limited sample size and cross-sectional design, our findings highlight that gut microbial composition and structure may be important for host adaptation to cold environments and may also serve as a useful reference for future studies on the adaptive mechanisms and conservation strategies of endangered herbivores, including the Mongolian wild ass.

## 1. Introduction

The Mongolian wild ass (*Equus hemionus hemionus*), belonging to the family Equidae and the genus *Equus*, is a subspecies of the Asiatic wild ass (*Equus hemionus*) and serves as an important flagship species of the desert-steppe ecosystems in Asia, playing a crucial role in maintaining regional ecological balance [1]. However, due to habitat fragmentation and increasing human disturbances, its population has declined sharply [2,3]. This species has been listed as First-Class Protected Wildlife (the highest protection level under China’s Wildlife Protection Law) and is categorized as Near Threatened (NT) on the IUCN Red List of Threatened Species [2,4]. In the wild, Mongolian wild asses are confronted with harsh winter conditions characterized by extremely low temperatures and limited food resources, which may exert significant effects on their survival and adaptation. Although previous studies have revealed their behavioral adaptation strategies, such as migration and foraging preferences [5], the physiological mechanisms underlying these adaptations remain poorly understood, particularly the role of gut microbiota in coping with energy and nutritional constraints during the cold season.

Gut microbiota, often referred to as the host’s “second genome” [6], play essential roles in energy metabolism, immune regulation, and the maintenance of intestinal homeostasis, thereby serving as an important physiological foundation for animals to withstand environmental stressors [7,8]. Research on model organisms has shown that the gut microbiota of mice can alleviate physiological stress caused by cold exposure by regulating energy utilization, and thereby enhancing host adaptation under low-temperature conditions [9,10]. This finding provides a theoretical basis for understanding the environmental adaptation of host–microbe interactions. In herbivores, this function is particularly prominent: gut microbiota decompose plant fibers and generate SCFAs, which provide energy to the host [11]. Under winter conditions, when food quality declines and resources are limited, this metabolic pathway becomes especially critical. For instance, in white-lipped deer, gut microbial communities enriched in SCFA-producing taxa, along with the activation of energy metabolism and immune pathways, have been shown to significantly enhance adaptation to cold and food scarcity [12].

Nevertheless, research on how the gut microbiota of Mongolian wild asses responds to dietary changes during the cold season remains scarce. As a typical desert-adapted species, the survival of Mongolian wild asses may heavily depend on structural and functional remodeling of gut microbial communities to mitigate the dual pressures of winter cold and high-fiber, low-nutrient diets. With the advancement of metagenomics, researchers are now able to comprehensively resolve both the taxonomic composition and functional characteristics of gut microbiota [13], providing new opportunities for in-depth exploration of the cold-season adaptation mechanisms in Mongolian wild asses. Therefore, this study aims to employ metagenomic approaches to systematically analyze the composition and functional features of the gut microbiota of Mongolian wild asses during the cold season, and to investigate their associations with dietary plant composition. The findings are expected not only to fill existing gaps in microbial ecological research on this species but also to provide molecular evidence for understanding the adaptive mechanisms of endangered animals in extreme environments. Furthermore, this research will offer theoretical support for the development of microbiome-based conservation strategies, thereby contributing to population recovery and habitat management of Mongolian wild asses.

## 2. Materials and Methods

### 2.1. Sample Collection and Processing

Field sampling was conducted in June and November 2024 in the desert-steppe habitat of Urad Middle Banner, Bayannur City, Inner Mongolia, China. Fresh fecal samples of Mongolian wild asses were collected using a non-invasive approach. To minimize cross-contamination from environmental DNA, sampling was performed under conditions without precipitation or strong wind (≥10 m/s) within 24 h prior to collection. Samples were taken immediately after observing the defecation behavior of individuals through binoculars. Fresh feces were identified by the following criteria: dark brownish-green glossy surface, grass-green interior, and moist and soft texture. To ensure sample independence, spatial isolation (≥100 m between sites) and visual confirmation of separate defecation events were applied. Approximately 5 g of fecal material from the interior (avoiding ground contact) was collected with sterile disposable gloves and stored in sterile cryovials. Samples were snap-frozen in liquid nitrogen on site and transported to −80 °C for long-term storage, following standard protocols in microbiome research to ensure sample integrity.

### 2.2. Metagenomic Sequencing and Annotation

Fecal DNA was extracted using the HiPure Universal DNA Kit (Genepioneer, Nanjing), with integrity verified by 1% agarose gel electrophoresis, and quantity and purity assessed by Qubit and Nanodrop. DNA was sheared to ~300 bp using a Covaris M220 ultrasonicator and paired-end (PE) libraries were prepared using the TruSeq™ DNA Sample Prep Kit. Libraries were PCR-amplified and sequenced on the Illumina NovaSeq 6000 platform with PE150 mode (Q30 ≥ 90%). Raw reads were quality-controlled with fastp (v0.20.0, parameters: -q 5 -n 5), followed by host sequence removal via Bowtie2 (v2.3.5.1) alignment against the Mongolian wild ass genome (GCF_041296235.1). On average, ~12.11 Gb of high-quality data were obtained per sample. Clean reads were assembled using MEGAHIT (v1.2.9, -min-contig-len 500, and -presets meta-large). Open reading frames (ORFs) were predicted from assembled contigs (≥500 bp) using Prodigal (v2.6.3, parameters: -p meta -m). Redundancy removal was performed with CD-HIT (v4.8.1, -c 0.95 -aS 0.9 -g 1 -r 1 -d 0) to generate a non-redundant set of Unigenes. Clean reads were then mapped to Unigenes using bwa (v0.7.17), and genes with <2 mapped reads per sample were filtered out to form the final non-redundant gene catalog.

Taxonomic annotation was conducted using Kraken (v2.1.2) based on k-mer alignment, with databases covering bacteria, archaea, fungi, and viruses. A confidence threshold of 0.2 was applied to ensure reliable taxonomic assignments. Abundance and gene count tables were generated at multiple taxonomic levels (domain to species) for downstream analyses. KEGG functional annotation was performed using DIAMOND (v2.0.6.144, blastp, e-value ≤ 1 ×10^−5^), resulting in KO entry abundance and functional gene distribution profiles.

### 2.3. trnL-Based Plant Composition Sequencing and Annotation

For dietary plant composition analysis, the DNA solutions obtained in Section 2.2 were directly used for trnL chloroplast DNA metabarcoding, with the trnL_g-h region amplified using universal primers trnL_g (5′-GGGCAATCCTGAGCCAA-3′) and trnL_h (5′-CCATTGAGTCTCTGCACCTATC-3′). PCR was conducted in a 20 μL reaction containing 4 μL 5× FastPfu Buffer, 2 μL 2.5 mM dNTPs, 0.8 μL of each primer (5 μM), 0.4 μL FastPfu Polymerase, and 10 ng template DNA. The PCR program included an initial denaturation at 95 °C for 5 min, followed by 30 cycles (95 °C for 30 s, 58 °C for 30 s, 72 °C for 45 s), and a final extension at 72 °C for 10 min. Each sample was amplified in triplicate with negative controls included. PCR products (~600 bp) were confirmed by 2% agarose gel electrophoresis and purified using the AxyPrep DNA Gel Extraction Kit. Quantification was performed using the QuantiFluor™-ST fluorescence system, and libraries were constructed with the NEXTFLEX Rapid DNA-Seq Kit (Bioo Scientific, USA). Sequencing was carried out on the NovaSeq 6000 platform with paired-end 300 bp reads (Q30 ≥ 90%).

Bioinformatics analysis workflow included: (1) quality control with Trimmomatic (v0.30); (2) merging paired-end reads using FLASH (v1.2.7); (3) denoising with the DADA2 method in the QIIME 2 platform to generate high-resolution amplicon sequence variants (ASVs); (4) taxonomic annotation of ASVs via BLASTn (e-value ≤ 1 ×10^−10^) against the NCBI nt database.

### 2.4. Data Analysis

All data analyses were conducted in R (version 4.3.2). Group differences were tested with the Wilcoxon rank-sum test, with *p*-values corrected using the Benjamini–Hochberg method. The vegan package was used to calculate α- and β-diversity indices of gut microbiota. α-diversity included the Shannon index (influenced by both richness and evenness and particularly sensitive to rare taxa), the Simpson index (which places greater weight on dominant groups), and Faith’s phylogenetic diversity (PD, which measures diversity based on phylogenetic branch lengths). Group differences were assessed statistically. β-diversity analysis was based on Bray–Curtis distances calculated with vegan, followed by PCoA. Community structure visualizations were generated using ggplot2. Statistical significance of group differences was assessed by permutational multivariate analysis of variance (PERMANOVA) with 999 permutations in vegan.

Differential abundance analysis of KEGG Orthology (KO) entries was conducted using FMAP (Functional Mapping and Analysis Pipeline, v0.13), with significantly different KOs (*p* < 0.05) mapped to KEGG level 3 pathways. Functional enrichment trends were visualized using ggplot2. To evaluate the correlations between dietary plant composition (family level) and gut microbiota composition (phylum level), as well as between their respective α-diversity indices (Shannon, Simpson, and PD), Spearman’s rank correlation coefficients were calculated using the dplyr package, with significance testing performed. Correlation heatmaps were generated using ggplot2 and ggpubr.

## 3. Results

**Figure 1:**
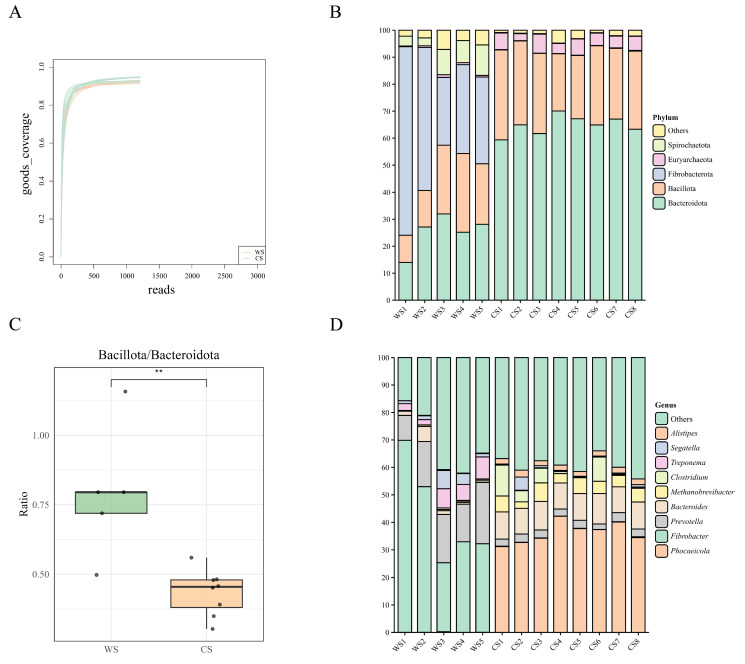
Gut Microbial Diversity, Dominant Taxa, and B/B Ratio in Mongolian Wild Asses Across Seasons. (**A**) Rarefaction curves of Good’s coverage for each sample. The curves plateaued near 1.0, indicating sufficient sequencing depth and comprehensive coverage of microbial diversity. (**B**) Relative abundance of dominant bacterial phyla (>1%) in each sample. (**C**) Bacillota to Bacteroidota (B/B) ratio comparison across the WS and CS. ** indicates *p* < 0.01. (**D**) Relative abundance of dominant bacterial genera (>1%) in each sample.

A total of 13 fecal samples were collected in this study, including 5 samples in June (defined as the warm-season group, WS group) and 8 samples in November (defined as the cold-season group, CS group). Metagenomic sequencing of these 13 Mongolian wild ass fecal samples generated 157,777.97 Mbp of raw data, with an average of 121,367.67 Mbp per sample. After quality control, 157,390.27 Mbp (99.75%) of clean data were obtained, averaging 12,106.94 Mbp per sample. Across the 13 samples, 22,830,747 coding sequences (CDS) were predicted. After redundancy removal, a total of 15,980,593 non-redundant genes were retained, including 5,958,601 genes in the WS group and 10,234,434 genes in the CS group. Good’s coverage analysis indicated that sequencing coverage rapidly approached 100% with increasing sequencing depth and plateaued at approximately 1000–1500 reads, suggesting that sequencing depth was sufficient to capture the majority of detectable microbial taxa and had reached saturation (Figure 1A).

### 3.1. Gut Microbiota Composition and Abundance Analysis

Based on the metagenomic sequencing data, this study selected the WS and CS groups of samples with taxonomic annotation results of the gut microbiota at the phylum and genus levels and calculated the relative abundance of each taxon. Dominant taxa with relative abundance exceeding 1% were identified, and stacked bar plots were generated to visualize the distributions across samples (Figure 1B,D). At the phylum level, the dominant groups were Bacteroidota (49.61%), Bacillota (24.90%), Fibrobacterota (16.46%), Euryarchaeota (3.31%), and Spirochaetota (2.81%). In the WS group, dominant phyla included Fibrobacterota (42.59%), Bacteroidota (25.28%), Bacillota (20.10%), Spirochaetota (7.08%), Thermodesulfobacteriota (1.52%), and Actinomycetota (1.16%). In the CS group, Bacteroidota (64.81%), Bacillota (27.90%), and Euryarchaeota (4.97%) were predominant.

At the genus level, dominant taxa included *Phocaeicola* (22.34%), *Fibrobacter* (16.46%), *Prevotella* (7.78%), *Bacteroides* (6.83%), *Methanobrevibacter* (3.06%), *Clostridium* (2.48%), *Treponema* (2.00%), *Segatella* (1.71%), and *Alistipes* (1.30%). In the WS group, dominant genera were *Fibrobacter* (42.59%), *Prevotella* (15.81%), *Treponema* (5.02%), *Segatella* (2.87%), and *Bacteroides* (1.92%). In the CS group, the dominant taxa included *Phocaeicola* (36.24%), *Bacteroides* (9.89%), *Methanobrevibacter* (4.70%), *Clostridium* (3.88%), *Prevotella* (2.77%), *Alistipes* (2.02%), and *Solibaculum* (1.38%).

Wilcoxon rank-sum tests with Benjamini–Hochberg correction revealed significant group differences. At the phylum level, Bacteroidota and Euryarchaeota were significantly lower in the WS group compared to the CS group (*p* < 0.01), whereas Fibrobacterota, Spirochaetota, Thermodesulfobacteriota, and Actinomycetota were significantly higher in the WS group (*p* < 0.01). Moreover, the B/B ratio, which reflects host energy metabolism strategies and environmental adaptability [14,15], was significantly higher in the WS group than in the CS group (*p* = 0.0031, Figure 1C). At the genus level, *Phocaeicola*, *Bacteroides*, *Methanobrevibacter*, *Clostridium*, *Alistipes*, and *Solibaculum* were significantly lower in the WS group, whereas *Fibrobacter*, *Prevotella*, *Treponema*, and *Segatella* were significantly higher (all *p* < 0.05).

### 3.2. Gut Microbial Community Structure

**Figure 2:**
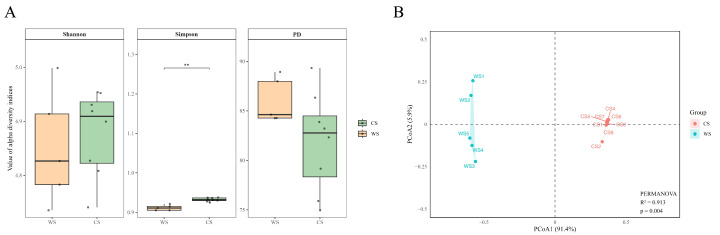
Seasonal Variation in Gut Microbiota Diversity and Community Structure of Mongolian Wild Ass. (**A**) α-Diversity of gut microbiota, including Shannon, Simpson, and Faith’s PD indices. Boxplots show the distribution within each seasonal group. ** indicates *p* < 0.01 (Wilcoxon test with FDR correction). (**B**) PCoA of gut microbial communities based on Bray–Curtis dissimilarity. Each point represents a sample, colored by group (WS and CS). Shaded ellipses indicate 95% confidence intervals. Group separation was evaluated by PERMANOVA (R^2^ = 0.913, *p* = 0.004).

To comprehensively assess gut microbial α-diversity at different stages, three indices were calculated at the species level: Shannon, Simpson, and PD. Results showed differences across groups, with the Simpson index displaying a highly significant difference (*p* < 0.01), while Shannon and PD showed no significant variation (Figure 2A). PCoA based on Bray–Curtis distances (Figure 2B) revealed clear separation between WS and CS groups, with CS samples clustering tightly and WS samples more dispersed. PERMANOVA further confirmed significant differences in community structure (R^2^ = 0.913, *p* = 0.004), indicating that grouping explained 91.3% of community variation.

### 3.3. KEGG Functional Analysis

Functional annotation of the non-redundant gene catalog using the KEGG database identified six major KEGG level 1 categories, which represent the broadest functional classifications. Among these, “Metabolism” was the most dominant (58.35% ± 0.38%), followed by “Genetic Information Processing” (13.76% ± 0.60%), “Cellular Processes” (9.10% ± 0.89%), “Environmental Information Processing” (8.46% ± 0.26%), “Human Diseases” (6.69% ± 0.29%), and “Organismal Systems” (3.63% ± 0.10%). At KEGG level 2, which provides more specific functional groupings within each major category, 47 pathways were detected, with 22 pathways exceeding 1% relative abundance, of which 19 differed significantly between groups (Table 1).

**Figure 3:**
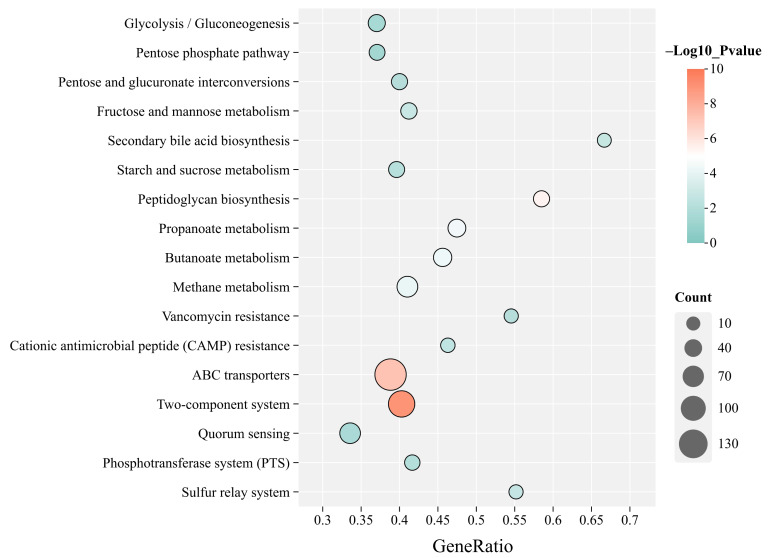
KEGG Level 3 pathway enrichment of significantly upregulated genes in the cold season. The *y*-axis represents KEGG Level 3 pathway names, while the *x*-axis indicates the proportion of upregulated genes within each pathway. Bubble size corresponds to the number of genes enriched in each pathway, and bubble color denotes statistical significance as −Log10(*p* value). All displayed pathways are significant (*p* < 0.05).

At KEGG level 3, which offers the most detailed pathway-level resolution, enrichment analysis was performed, and pathways that were both significant and predominantly upregulated in the cold season (i.e., the number of upregulated genes exceeded that of downregulated genes) were selected. These pathways were visualized using a KEGG bubble plot (Figure 3) to investigate the functional adaptations of the gut microbiota in Mongolian wild asses during the cold season. The results showed that a total of 17 pathways were identified, of which 10 could be classified as metabolic pathways. Specifically, ABC transporters (152 genes upregulated), Two-component system (111 genes upregulated), Methane metabolism (65 genes upregulated), Quorum sensing (64 genes upregulated), Butanoate metabolism (45 genes upregulated), and Propanoate metabolism (41 genes upregulated) were among the most enriched.

### 3.4. trnL-Based Plant Composition

**Figure 4:**
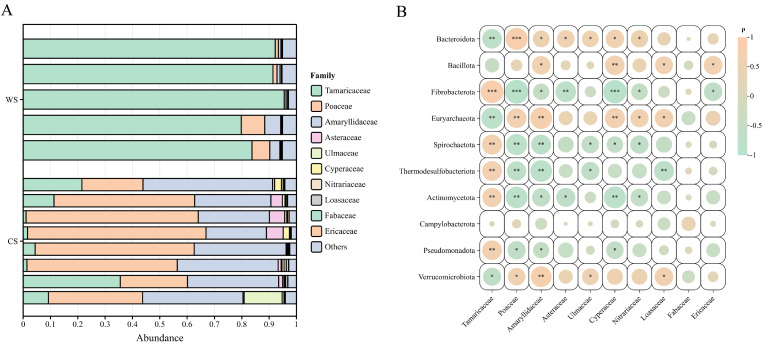
Seasonal Dietary Plant Composition and Its Associations with Gut Microbial Phyla in Mongolian Wild Asses. (**A**) Stacked bar plot of plant family composition in the diet of Mongolian wild asses across seasons. (**B**) Spearman correlation heatmap between plant families in the diet and gut microbial phyla. The heatmap illustrates Spearman’s rank correlations (ρ) between plant families in the diet (*x*-axis) and gut microbial phyla (*y*-axis) in Mongolian wild asses. The color gradient represents the strength and direction of correlations, with pink indicating positive and green indicating negative correlations. The size of the circle in each cell reflects the significance level of the correlation. * indicates *p* < 0.05, ** indicates *p* < 0.01, and *** indicates *p* < 0.001; blank cells are not statistically significant.

The dietary plant composition of Mongolian wild ass was assessed by high-throughput sequencing of the chloroplast trnL intron g–h fragment, a widely used plant DNA barcode for herbivore diet analysis due to its short length, high copy number, and broad taxonomic coverage in fecal samples [16,17]. Results indicated seasonal differences in plant composition: in the warm season, dominant families were Tamaricaceae (88.54%), Poaceae (3.68%), and Amaryllidaceae (2.43%); in the cold season, predominant detected families included Poaceae (46.80%), Amaryllidaceae (33.00%), Tamaricaceae (10.74%), Asteraceae (2.50%), Ulmaceae (1.85%), and Cyperaceae (1.06%) (Figure 4A). Wilcoxon rank-sum tests with BH correction showed that Tamaricaceae was significantly higher in the WS group (*p* = 0.0104), while Poaceae, Amaryllidaceae, Asteraceae, Ulmaceae, Cyperaceae, Nitrariaceae, and Loasaceae were significantly higher in the CS group (*p* < 0.05).

Spearman correlation analysis between the top 10 abundant gut microbiota (phylum level) and dietary plant composition (family level) indicated positive correlations for Bacteroidota, Bacillota, Euryarchaeota, and Verrucomicrobiota with dominant detected plant families, while Fibrobacterota, Spirochaetota, Thermodesulfobacteriota, Actinomycetota, and Pseudomonadota showed predominantly negative correlations, some of which were significant (*p* < 0.05) (Figure 4B). Alpha diversity analysis of plant composition showed that Simpson and Shannon indices were significantly higher in the CS group compared to the WS group (*p* < 0.01). However, no significant correlations were detected between plant composition diversity and gut microbiota diversity (*p* > 0.05).

## 4. Discussion

### 4.1. Cold Adaptation of Gut Microbiota Composition and Structure

The results of this study showed that during the cold season, the relative abundance of Bacteroidota and Euryarchaeota in the gut of the Mongolian wild ass was significantly higher than in the warm season. Bacteroidota are primarily involved in degrading complex carbohydrates such as polysaccharides and cellulose [18], and their dominance during the cold season—when crude fiber intake increases—enhances the host’s ability to utilize low-quality forage. Furthermore, the B/B ratio of the Mongolian wild ass was significantly higher in the warm season than in the cold season, which is easily explained. A higher B/B ratio is generally associated with greater capacity for energy intake and storage, typically observed during the lush summer pasture season, thereby facilitating energy accumulation to cope with winter food scarcity. In contrast, a lower B/B ratio is often linked to enhanced fiber-degrading capacity, more suitable for arid and cold seasons, enabling efficient utilization of crude fiber to sustain basal metabolism [19]. However, some studies (e.g., in *Lasiopodomys brandtii* [20] and *Ovis aries* [21]) have reported that cold stress may lead to an increased B/B ratio, suggesting that such changes may help maintain metabolic balance under cold conditions. This discrepancy suggests that gut microbiota adaptation to cold stress may be species-specific and context-dependent.

At the genus level, *Bacteroides* and related genera (e.g., *Phocaeicola*, *Alistipes*) were significantly enriched in the cold season. This pattern is consistent with observations in Burmese pythons(*Python molurus*) and brown bears(*Ursus arctos*), in which the abundance of *Bacteroides* increases under starvation or prolonged fasting conditions [22,23,24]. Such enrichment may stem from their ability to degrade host-derived mucin glycans, allowing them to maintain community stability and provide energy to the host when dietary polysaccharides are insufficient. This supports host metabolism and physiological demands under low temperatures and declining food quality. The increase in *Alistipes* in the cold season also holds important ecological significance. This genus not only participates in SCFA metabolism—promoting the production of butyrate and other SCFAs under cold exposure or huddling conditions to aid in energy conservation and thermoregulation [25]—but also acts as a bile acid-tolerant taxon along with *Bacteroides* [26], thereby enhancing lipid metabolism through the regulation of acetate production [27]. In addition, *Bacteroides* can modulate T-cell differentiation, suppress inflammatory responses, and enhance host immunity by secreting polysaccharide A [28,29], while *Alistipes* may also contribute to immune responses via butyrate and other metabolic products [30]. Taken together, the functional enrichment of Bacteroidota and their key dominant genera during the cold season reflects comprehensive microbial adaptation strategies in energy acquisition, metabolic regulation, and immune modulation, thereby enabling the host to maintain physiological homeostasis and enhance survival under low-temperature and nutrient-limited conditions.

Previous studies have indicated that higher α-diversity is usually associated with more complex and stable gut microbial communities, which not only strengthen the host’s resistance to external disturbances but also enhance its adaptive potential under environmental fluctuations. Conversely, reduced or lost α-diversity is closely linked to various diseases [31,32]. In this study, the Simpson index of Mongolian wild asses was significantly higher in the cold season compared to the warm season, while the Shannon index showed an increasing trend but without significant differences. This pattern may be related to the marked enrichment of dominant taxa such as Bacteroidota in winter, while differences in rare taxa between the two groups were relatively small. These results suggest that Mongolian wild ass may increase the abundance of core microbiota, enhancing intestinal microbial diversity in winter to maintain community stability and functional diversity, which may help them better adapt to low temperatures and declining forage quality. Similar seasonal changes were observed in free-ranging Tibetan macaques (*Macaca thibetana*), whose α-diversity in winter was significantly higher than in other seasons, facilitating more efficient energy acquisition from high-fiber plants [33]. Meanwhile, PCoA analysis revealed significant separation between CS and WS samples along the two principal coordinates, with tighter clustering of CS samples, indicating that cold environmental pressures drive convergence of microbial community structures and reduce inter-individual variation. Such integration and optimization of microbial communities may represent an important microecological strategy for the host to cope with extreme environments [12].

### 4.2. Cold-Season Functional Adaptations of Gut Microbiota

Functional prediction results further indicated that the metabolism-related functions of the Mongolian wild ass gut microbiota are dominant, accounting for a relative abundance of 58.35%, highlighting the central role of gut microbes in the digestion, absorption, and metabolism of nutrients in the host [11]. KEGG functional analysis revealed significant seasonal differences in microbial functional profiles between the cold and warm seasons, a phenomenon also reported in wild animals such as giant pandas (*Ailuropoda melanoleuca*) [34], Chinese alligators (*Alligator sinensis*) [35], and white-faced capuchins (*Cebus capucinus imitator*) [36]. In KEGG Level 3 functional enrichment analysis, several key metabolic pathways were significantly upregulated during the cold season, including ABC transporters, Two-component systems, and Quorum sensing. ABC transporters facilitate the active transport of substrates such as sugars and amino acids via ATP hydrolysis [37], potentially enhancing nutrient absorption efficiency under low temperature and food scarcity to meet host energy demands [38]. Two-component systems help microbes rapidly sense environmental changes and modulate stress responses [39], possibly maintaining gut metabolic homeostasis under cold and nutrient-limited conditions. The upregulation of Quorum sensing suggests a more active microbial population sensing in the cold season, thereby regulating interspecies interactions that influence host immunity and gut functional stability. Notably, the significant enhancement of butanoate and propanoate metabolism pathways indicates increased production of SCFAs in the cold season. SCFAs not only serve as important energy substrates for the host but may also contribute to thermogenesis and metabolic regulation, helping maintain body temperature and energy balance [25]. It is important to note that metagenomic analysis reveals the functional potential of the gut microbiome based on gene content, rather than confirming actual gene expression or metabolite production. Therefore, these results should be complemented with meta-transcriptomics or metabolomics to verify the functional activity of the identified pathways.

### 4.3. trnL-Based Plant Composition and Seasonal Feeding

According to the ERA5 dataset [40], the average monthly precipitation in Urad Middle Banner, Bayannur, Inner Mongolia, China, was only 11.62 mm from January to June 2024, but increased significantly to 105.25 mm in August and September. Because the rainy season had not yet begun and herbaceous plants such as Poaceae were still immature in early summer, the Mongolian wild ass likely exhibited a substantially reduced grazing frequency on Poaceae. Previous studies have indicated that under arid conditions, Mongolian wild asses often feed on vegetation near water sources, such as Tamaricaceae plants [41]. Other research has shown that Mongolian wild asses prefer the young shoots of Tamaricaceae plants, such as *Reaumuria soongorica* [42]. This may explain the high proportion of Tamaricaceae consumption (up to 88.54%) observed in this study during the warm season (June). However, this hypothesis still requires further verification through investigations of plant availability and phenology.

In winter, Mongolian wild asses primarily feed on Poaceae (54.74%), followed by Chenopodiaceae (14.96%) and Tamaricaceae (9.48%) [43], which is largely consistent with the results of this study, indicating that Poaceae and Tamaricaceae are consistent and potentially key food sources during the cold season. Poaceae generally have higher nutritional value, better digestibility, and greater palatability than shrubs or needle-leaved plants, which may contribute to higher consumption rates [42,44]. In addition, this study also detected consumption of Amaryllidaceae, Asteraceae, and Ulmaceae plants during the cold season, which have rarely been reported in previous studies, suggesting that the feeding range of Mongolian wild asses may be broader than previously expected. It is important to emphasize that the diet of wild animals reflects not only their preferences but is also strongly influenced by food availability. Mongolian wild asses may select high-nutritional-value plants when resources are abundant, but adopt a more generalized diet under resource scarcity [44]. This phenomenon may explain our observation that α-diversity of dietary plant composition was significantly higher in winter than in summer, indicating that plant composition diversity expands under cold and resource-limited conditions, which may reflect a potential adaptive response to seasonal food shortages.

Notably, a study on the gut microbiota of 33 large herbivores in the semi-arid savannas of East Africa showed that food diversity did not significantly affect gut microbiota diversity. However, food composition was significantly associated with gut microbial composition, and seasonal differences in food composition explained 25% of cross-species microbial seasonality [45]. This is consistent with the findings of this study: although Mongolian wild asses exhibited significantly increased plant composition diversity in the cold season, no significant correlation was detected between plant composition diversity and gut microbial diversity. This suggests that factors influencing gut microbial community structure are not limited to food types but may also include temperature, metabolic status, and host physiological regulation. Furthermore, this study found that Bacteroidota, Bacillota, Euryarchaeota, and Verrucomicrobiota were positively correlated with the predominant detected plant families, suggesting that these taxa could contribute to the degradation of cellulose, polysaccharides, and other plant secondary metabolites, which may facilitate host utilization of the high-fiber, low-soluble-sugar diet dominated by Poaceae and Amaryllidaceae in winter. Conversely, Fibrobacterota, Spirochaetota, Thermodesulfobacteriota, Actinomycetota, and Pseudomonadota showed mainly negative correlations with plant composition, possibly reflecting their reduced dependence on specific substrates or environmental conditions under cold conditions.

In summary, this study systematically characterized the seasonal variations in gut microbiota composition and the functional potential of Mongolian wild asses and their associations with dietary plant composition, providing insights into potential microbial adaptations to cold and low-quality food conditions. Several limitations should be acknowledged. The sample size was constrained by field conditions and conservation regulations, and cold-season samples were collected in November rather than during peak winter, potentially underestimating full cold-stress responses. This study’s cross-sectional design and lack of longitudinal monitoring limit the assessment of inter-annual variation and individual dietary plasticity. Additionally, the use of the trnL marker provides only a proxy for dietary plant composition, and the Wilcoxon rank-sum test, though commonly applied, does not fully account for the compositional nature of relative abundance data. Despite these constraints, this work offers a valuable baseline for understanding how gut microbiota may support energy extraction, fiber degradation, and metabolic homeostasis under seasonal resource fluctuations. These findings highlight the fact that the gut microbial composition and structure may be important for host adaptation to cold environments and may also serve as a useful reference for future studies on the adaptive mechanisms and conservation strategies of endangered herbivores, including the Mongolian wild ass. Future studies integrating larger sample sizes, multi-season and multi-population sampling, and multi-omics approaches (e.g., metatranscriptomics and metabolomics) will be essential to fully elucidate the dynamic roles of gut microbiota in host adaptation.

## Figures and Tables

**Table 1:** Relative abundance (%) of KEGG Level 2 pathways (>1%) in WS and CS groups, including Wilcoxon rank-sum test BH-adjusted *p*-values.

KEGG Level 2 Pathways	WS (Mean)	WS (SD)	CS (Mean)	CS (SD)	Wilcoxon _adj.p
Carbohydrate metabolism	14.2188	0.2585	14.1086	0.0982	0.5486
Amino acid metabolism	7.8327	0.3125	8.5900	0.0774	0.0021
Glycan biosynthesis and metabolism	8.3952	0.2417	7.1323	0.1417	0.0021
Metabolism of cofactors and vitamins	5.9110	0.0531	5.8319	0.0520	0.0342
Replication and repair	5.7053	0.2280	5.6533	0.1044	0.9433
Energy metabolism	4.5317	0.0714	5.4279	0.0960	0.0021
Translation	3.9471	0.0972	4.8214	0.1235	0.0021
Nucleotide metabolism	4.2836	0.1373	4.7683	0.0543	0.0021
Cellular community—prokaryotes	4.7670	0.1461	3.8899	0.0393	0.0021
Signal transduction	5.0111	0.2804	3.9948	0.0834	0.0021
Membrane transport	3.6382	0.0994	4.3342	0.1052	0.0021
Lipid metabolism	3.9895	0.1303	4.0340	0.0645	0.5486
Biosynthesis of other secondary metabolites	3.2261	0.0751	2.9493	0.0421	0.0021
Folding, sorting and degradation	2.8743	0.0424	2.9547	0.0207	0.0080
Metabolism of other amino acids	2.6941	0.0472	2.8986	0.0502	0.0021
Cell growth and death	2.5095	0.0350	2.3095	0.0484	0.0021
Drug resistance: antimicrobial	2.3286	0.0686	2.5330	0.0311	0.0021
Transport and catabolism	1.9091	0.1134	1.5829	0.0594	0.0021
Metabolism of terpenoids and polyketides	1.9260	0.1857	1.5668	0.0179	0.0021
Infectious disease: bacterial	1.4953	0.0379	1.4124	0.0159	0.0021
Xenobiotics biodegradation and metabolism	1.0368	0.0239	1.2379	0.0204	0.0021
Endocrine system	1.0108	0.0350	1.0598	0.0223	0.0342

## Data Availability

The raw sequence data reported in this paper have been deposited in the Genome Sequence Archive (Genomics, Proteomics & Bioinformatics 2021) in National Genomics Data Center (Nucleic Acids Res 2022), China National Center for Bioinformation/Beijing Institute of Genomics, Chinese Academy of Sciences (GSA: CRA030274), and are accessible at https://ngdc.cncb.ac.cn/gsa (accessed on 26 September 2025).
